# ‘To Know That You Are a Link in the Chain’: A Realist Evaluation to Explore How Digital, Intensive, Parent‐Implemented Interventions Work for Children With Speech Sound Disorder, Why, and for Whom

**DOI:** 10.1111/1460-6984.70049

**Published:** 2025-05-16

**Authors:** Naomi Leafe, Emma Pagnamenta, Mark Donnelly, Laurence Taggart, Jill Titterington

**Affiliations:** ^1^ Institute of Nursing and Health Research Ulster University Belfast UK; ^2^ School of Psychology and Clinical Language Sciences University of Reading Reading UK; ^3^ School of Computing Ulster University Belfast UK

**Keywords:** digital health, intensive, parent‐implemented, realist methods, speech and language therapy, speech sound disorder

## Abstract

**Introduction:**

Children with moderate to severe speech sound disorder (SSD) need intensive therapy to increase intervention effectiveness and efficiency. However, worldwide speech and language therapists (SLTs) report that it is difficult to implement recommended intervention intensities in clinical practice. Supporting parents/carers to deliver home‐intervention, facilitated through digital tools, has the potential to circumvent these difficulties and increase practice intensity. This realist evaluation builds on our earlier realist review on intensive, digital, parent‐implemented interventions for children with SSD through exploring the experiences of stakeholders to optimally understand what might work best, for whom, and why in clinical practice.

**Methods:**

We undertook a realist evaluation to test and refine our initial programme theories developed in our earlier realist review through focus groups with key stakeholders. Five focus groups were conducted with SLTs (*n* = 22), and two focus groups with parents/carers of children with SSD aged 4–5 years (*n* = 6). A realist methodology approach was used to collect and analyse the data, including the development of context‐mechanism‐outcome configurations. Middle‐range theories of adult‐learning, self‐efficacy and parenting styles were used to develop our theoretical thinking.

**Results:**

Programme theories from the earlier realist review about how the intervention works were refined, refuted, or confirmed. The refined theories are presented across three areas to demonstrate the journey of engaging in a digital, intensive parent‐implemented intervention: (1) Readiness to engage; (2) Implementation of the intervention; and (3) Sustaining momentum. The theories offer insight into mechanisms that support and train families to engage in home‐practice through digital tools, including important contextual factors needing consideration in implementation.

**Conclusion:**

Digital, intensive, parent‐implemented interventions for children with SSD have the potential to improve the effectiveness and efficiency of SLT services in certain contexts and improve children's outcomes worldwide. Mechanisms of change, and impactful contexts at each point of the journey of involvement need consideration to successfully empower and support parents/carers and their children with SSD.

**WHAT THIS PAPER ADDS:**

*What is already known on this subject*

- Clinicians worldwide face challenges providing the optimal intensity of intervention for children with SSD. Studies have explored parent‐implemented interventions and digital tools to increase intervention intensity. These new and innovative service delivery models can be effective in certain circumstances; however, further exploration is required to understand why digital, parent‐implemented interventions may work, for whom, and how.

*What this study adds*

- This paper uses a realist methodology approach to capture stakeholder experiences to develop underpinning theories about digital, intensive, parent‐implemented interventions for children with SSD. The new theoretical insight from this realist evaluation builds upon our understanding of what makes this intervention work (or not), who it works for, and why.

*What are the clinical implications of this study?*

- SLTs and health services will have a clearer understanding of how to support parents/carers to implement intensive home‐intervention through digital tools, and which factors impact its effectiveness. New understanding demonstrates how digital tools to support parents/carers have the potential to support more intensive practice for children with SSD.

## 1 Background

### 1.1 Rationale for the Evaluation

Speech sound disorder (SSD) is a childhood communication disorder which impacts children's speech intelligibility and ability to communicate with other people (RCSLT 2024). Persistent SSD is associated with risk of immediate and long‐term impact on educational outcomes and social, emotional, and behavioural well‐being (Wren et al. 2023, 2021; RCSLT 2024). Appropriate and timely intervention is imperative to reduce this risk (Wren et al. 2023).

The intensity of speech and language therapy intervention that children with SSD receive is part of this picture. Optimal intervention intensity for SSD is not currently conclusive, partly due to reporting differences and the need for further clinical trials (Maas 2024). However, globally, it is generally reported that higher‐intensity intervention through more frequent sessions (dose frequency) and a greater number of trials per session (dose) generates better outcomes than lower‐intensity intervention (Allen 2013; Cummings et al. 2021; McFaul et al. 2022; Maas 2024). Children with moderate to severe motor or phonological SSD are recommended to receive two to four therapy sessions per week, with at least 70–100+ trials of a target sound per session (e.g., Allen 2013; Kaipa and Peterson 2016; Sugden et al. 2018; Cummings et al. 2021).

Optimal intervention is difficult to achieve in real‐world clinical settings (Maas 2024), impacted by contextual factors such as travel, resources, caseload sizes, fatigue, staff training and scheduling (Hegarty et al. 2018; Sugden et al. 2018; McFaul et al. 2022; Maas 2024). Globally, findings from studies investigating typical service delivery for children with SSD demonstrate evidence‐practice gaps (To et al. 2012; Brumbaugh and Smit 2013; Oliveira et al. 2014; Hegarty et al. 2018; Sugden et al. 2018). Children generally receive fewer sessions per week than recommended, typically receiving less than one session per week unless in specific educational settings (To et al. 2012; Oliveira et al. 2014; Hegarty et al. 2018; Sugden et al. 2018). Dose is generally under investigated, but where reported, it is lower than recommended (Kaipa and Peterson 2016; Hegarty et al. 2018).

Supporting parents/carers to deliver SSD intervention at home, alongside direct SLT input, could help increase the intensity of therapy practice. Studies exploring parent‐implemented interventions have found that parents/carers are able to deliver home‐intervention with adequate training and support (Flanagan and Ttofari Eecen 2018; Sugden et al. 2020, 2019; Sweeney et al. 2020; Sell et al. 2023). Parental confidence and self‐efficacy, role perceptions and expectations, and the parent/carer and SLT relationship can impact the effectiveness of this approach (Sugden et al. 2019; Leafe et al. 2025). Klatte et al. (2020) developed preliminary theories about collaborative practice between parent/carers and SLTs in a realist evaluation, outlining how factors such as developing shared understanding, co‐designing activities, and responding to parents/carers individual expectations can improve outcomes. The authors recognised that further research is needed, particularly through realist methods. This paper fills this gap by exploring these factors with stakeholders in the context of SSD.

Digital tools may provide a solution to service barriers as an intervention delivery platform, increasing service access and incentivising children and families to take part in intensive intervention at home in between direct sessions with their speech and language therapist (McKechnie et al. 2020; McLeod et al. 2020, 2023; Leafe et al. 2025). A recent effectiveness study found that a mobile game (SayBananas!), which provides children with SSD with high‐dose intervention with SLT oversight, can increase access to speech practice (McLeod et al. 2023). Children and families showed high engagement and motivation to practise, and there was a correlation between treated word progress and practise intensity. These findings suggest that mobile games alongside SLT support are a viable way to help increase intervention intensity. In contrast, McLeod et al.’s (2020) RCT compared an evidence‐based website (developed by McGill and McLeod 2019) to support parents/carers with strategies while waiting for SLT services to in‐person SLT intervention and an advice session. They found a significant difference between speech production outcomes for children who received in‐person sessions and the other two groups. When the same website was compared to a control group (McGill et al. 2020), no statistically significant difference was found between the two groups’ outcomes. These studies illustrate that specific active ingredients are needed for digital platforms to be effective in empowering parents/carers to support their children.

Leafe et al. (2024, 2025) earlier realist review used existing literature to explore potential active ingredients of digital, intensive, parent‐implemented interventions for children with SSD. Context‐mechanism‐outcome (CMO) configurations were used to show theoretical thinking about how digital, parent‐implemented and intensive interventions work, for whom, and why. See Table 1 for a glossary of key terms used in realist studies. Theories about the intervention (CMOs) were grouped into five programme areas: parent‐training, intervention intensity, partnership and collaboration, the SLT‐parent/carer‐child dynamic, and child‐participation. This subsequent realist evaluation aimed to further develop and refine theories from the earlier realist review and explore how the intervention would work in real‐world contexts, where difficulty implementing evidence‐based intensity is recognised.

**TABLE 1:** Glossary of key realist terminology.

Term	Definition
**Realist methodology**	Underpinned by realist philosophy, realist methods are informed by theory and use explanatory thinking to explore what makes interventions work, for whom, in what situations, and why (Hunter et al. 2022).
**Generative causation**	The idea that forces or mechanisms, which are often unobservable, trigger something to happen in certain contexts (Hunter et al. 2022).
**Retroduction**	A way of uncovering hidden forces or mechanisms that may not be visible that cause something observable to happen (e.g., empowerment or empathy) (Jagosh 2020; Hunter et al. 2022). Retroduction includes inductive and deductive thinking and theorising to understand the underlying factors that lead to certain outcomes in an intervention (Greenhalgh et al. 2017a).
**Programme theory **	A realist programme theory is an idea that explains the resources and responses of an intervention, explaining what works, who it works for, why, and what contexts contribute to the outcomes (Greenhalgh et al. 2017b).
**Middle‐range theory (MRT)**	A theory that can be applied broadly across different interventions in different contexts to explain how they work in implementation but can also be used to explain a specific intervention of focus (Greenhalgh et al. 2017b).
**Context **	The situation in which the intervention is delivered affects how or why the intervention mechanisms work or not.
**Mechanism **	Mechanisms are the forces that lead something to happen in an intervention, described as the resources the intervention offers and how people respond to the resources. Mechanisms are often unobservable and may be activated in different ways depending on the context the intervention is delivered in (Jagosh 2020).
**Outcome **	The result of an intervention through the interaction of contexts and mechanisms, which may be intended or unintended.
**Context‐mechanism‐outcome configurations (CMOs) **	CMO configurations are a framework or a tool that outline the key aspects that need explanation in realist methods, describing what works, for whom, how, and in which contexts. CMOs help to build theory by depicting the causal explanations of how contexts, mechanisms, and outcomes of an intervention react (Jagosh et al. 2015; Hunter et al. 2022).

### 1.2 Rationale for Using a Realist Evaluation Approach

A digital, intensive, parent‐implemented intervention for children with moderate to severe SSD is a complex intervention delivered in a complex system. It involves interacting components, behaviours and variability in outcomes depending on the person receiving the intervention, their worldview, the environment and surrounding policies (Pawson 2013). Theory‐based approaches, such as a realist approach, are beneficial in adding to knowledge, understanding and implementation of complex interventions (Pawson et al. 2004). Realist research offers insight into a programme's complexity by exploring mechanisms (the usually unobservable resources an intervention offers and responses of those involved), and relationships between mechanisms and contexts the intervention is delivered in (Hunter et al. 2022). This supports exploratory thinking about what makes an intervention work, in what situations, for whom, and why, aiming to uncover layers of reality that may not be perceivable on the surface (Pawson and Tilley 1997). The deep understanding of causality and impact of context on intervention outcomes arising from this evaluation, will underpin future development of a sustainable intervention in practice (Skivington et al. 2021).

### 1.3 Aims

#### 1.3.1 Aim

To evaluate, develop and refine underpinning theories on digital, parent‐implemented interventions for children aged 4–5 years with SSD through a realist evaluation using focus groups with SLTs and parents/carers of children with SSD.

#### 1.3.2 Objectives

1. To test underpinning programme theories developed through an earlier realist review for digital parent‐implemented interventions for children with SSD, using perspectives and experiences of stakeholders, so that theories can be accepted, refuted, or refined.
2. To further understand how contexts and mechanisms interact in the real world to produce certain outcomes, through gathering in‐depth insight from stakeholders.

The following research questions were considered: What are SLTs’ and parents/carers’ experiences with digital or parent‐implemented interventions for children with SSD? What makes these interventions work (or not)? Who do these interventions work for, and why? What contexts impact the effectiveness of digital, parent‐implemented interventions?

### 1.4 Environment Surrounding Evaluation

This evaluation is phase two of a three‐phase study to co‐produce a digital, intensive, parent‐implemented intervention for children with SSD. This evaluation aims to test and refine programme theories developed through an earlier realist review in phase one (Leafe et al. 2025). The underpinning theory developed in this realist evaluation will be used to co‐produce a digital tool to support intensive home‐practice for children with SSD (phase three).

### 1.5 Ethical Approval

Ethical approval for the study was obtained from the NHS Research Ethics Committee (Reference: 22/PR/0651; IRAS Project ID: 314024). Research governance was agreed and approved with Health and Social Care Trusts (HSCTs) involved in Northern Ireland (NI).

## 2 Methods and Procedures

This realist evaluation considers how the principles of supporting and training a parent/carer to implement intensive intervention for their child through a digital tool (app) could work when delivered as part of SLT services in the United Kingdom and beyond.

### 2.1 Design

This realist evaluation approach tested the programme theories developed in the earlier realist review (Leafe et al. 2025). Qualitative data was collected through focus groups with stakeholders to confirm, refute, or refine programme theories. RAMESES quality and reporting guidelines were followed in the design and reporting of this evaluation (Wong et al. 2016).

### 2.2 Recruitment and Sample Strategy

SLTs and parents/carers of children with SSD were recruited to provide insight into how the intervention may work in clinical practice.

#### 2.2.1 Speech and Language Therapists (SLTs)

SLTs with experience of working with children with SSD were recruited to take part. Four focus groups were conducted with Northern Ireland (NI) SLTs, and one group with SLTs from across the United Kingdom. In total, 22 SLTs participated. For recruitment in NI, a local collaborator from four of the NHS’ Health and Social Care Trusts (HSCTs) (Northern Trust, Southern Trust, South‐Eastern Trust, and Western Trust) disseminated a flyer and information sheet to potential participants in their teams. SLTs from across the UK were recruited on SLT social media sites and professional networks through a flyer.

#### 2.2.2 Parents/Carers

Parents/carers were recruited from the same four HSCTs in NI. They were included if their child: (1) had SSD as their main communication need; (2) was aged between 4 and 5 years at the time of the study or when they received SLT support for their speech; (3) was receiving or had received SLT support in an NI HSCT; and (4) had no other developmental, sensory, or physical diagnosis. Parents/carers were asked to provide their postcode to try to include a range of socioeconomic backgrounds. The local collaborator in each trust disseminated information sheets to SLTs to give to eligible parents/carers on their caseloads. To further support recruitment, a flyer was shared on social media platforms (e.g., X, Facebook groups). All parents/carers interested in participating were asked to contact the first author (NL) directly. Two focus groups were conducted with six parents/carers from HSCTs in NI.

### 2.3 Participants

A total of seven focus groups were conducted: four involving SLTs in NI (*n* = 17), one with SLTs working across the United Kingdom (*n* = 5), and two with parents/carers of children with SSD in NI (*n* = 6).

SLTs ranged from 2 to 36 years of clinical experience, summarised in Table 2. The four NI HSCTs involved in the study were represented by the SLTs involved. SLTs from outside NI worked within NHS settings, higher education institutes, or independently to support children in mainstream schools across the United Kingdom. Parent/carers represented the four NI HSCTs involved in this study. A total of 13 parents/carers had consented; however, two cancelled before the group, three were unable to find a suitable date, and two did not respond to invitations to join.

**TABLE 2:** Frequency table for number of years of experience of SLT participants.

Number of years experience	Frequency (*n* = 22)	Percentage
0–5 years	3	13.6%
6–10 years	4	18.2%
11–15 years	1	4.5%
16–20 years	3	13.6%
21–25 years	6	27.3%
26–30 years	1	4.5%
31–35 years	2	9.0%
36–40 years	2	9.0%

Abbreviation: SLT, speech and language therapist.

### 2.4 Data Collection Methods

A variety of data collection methods can be used in realist evaluation methodologies to develop programme theories. In this study, we used focus groups to help elicit deep stakeholder insight and added a layer of depth to discussion, described by Manzano (2022) as group reasoning. Focus groups were held on Zoom and lasted 2 h each, conducted by the first author (NL) and facilitated by a second member of the research team (JT or EP). All focus groups were audio and video recorded through Zoom and transcribed verbatim by NL.

Realist interviews are driven by theory, designed using techniques to test and refine the underpinning causal theories of an intervention (Pawson 1996; Pawson and Tilley 1997; Manzano 2016; Rees et al. 2024). Focus group questions were posed for the purpose of theory gleaning, refining, and/or consolidation (Manzano 2016; Rees et al. 2024). A teacher‐learner cycle helped consolidate theory, which is a dynamic process where the researcher shares theory ideas with participants (teacher to learner), who comment on the ideas based on their experiences to refine and add insight to the theory (i.e., participant becomes teacher, and researcher becomes learner) (Pawson 1996; Manzano 2016; Rees et al. 2024). Participants were presented with theories from the realist review, and asked whether they agreed, disagreed, or thought the theory needed refining. This supported in depth‐discussion between participants. Techniques such as recasting, and probing were used to uncover underlying insight from their experiences. Example questions are shown in Table 3.

**TABLE 3:** Example of realist interview questions used in the focus groups.

1	What do you think it is about somebody feeling that they don't know what they're doing that might mean that they don't want to practise at home?
2	Do you think there's something around a parent coming in with an expectation then, that somebody's going to help fix them, that maybe impacts on how well they engage in intervention—has anyone got any thoughts on that?
3	I wonder whether we could try and unpick that a little bit more, what do we think it is about an explanation—you say that it can become too complicated …what's the impact of that?

### 2.5 Data Analysis

Data was analysed by NL using realist coding and analysis techniques (Pawson and Tilley 1997; Pawson 2013; Wong et al. 2016). Programme theories form the unit of analysis in realist methods (Pawson 2013), and analysis is both deductive and inductive (top down and bottom up). Data was mapped to the existing context‐mechanism‐outcome configurations (CMOs) from the realist review. New insight from focus groups was used to refine and develop CMOs. Behaviour change theories offered a conceptual model, where the theoretical domains framework (TDF) (Atkins et al. 2017) provided a middle‐range (over‐arching) theoretical framework for analysis^1^. The domains were used to expand explanations and organise and interpret data. Existing theory was applied to groups of CMOs to add depth to thinking. Theories around self‐efficacy, adult‐learning, and parenting style were relevant to parents/carers involved in the intervention. Teaching style theories related to SLT involvement. These existing, middle‐range theories will be referred to in the findings and discussion.

Analysis began informally during data collection, where questions were adapted depending on emerging data (Rees et al. 2024). NVivo software was used as a tool for handling and organising data when more formal data analysis was employed, adapted from an approach used by Dalkin et al. (2020). Nodes were created for the five overarching programme areas from the realist review, then child nodes (sub‐nodes) for CMO titles, and further nodes for different data sources (e.g., SLT focus group). Focus group transcriptions were imported, and researcher reflections (from conducting focus groups, transcription, and analysis) were added to memos. Data extracts from transcriptions and memos were coded to nodes and child nodes. New nodes were created if novel thinking emerged.

Microsoft Word was used to create a working document between the research team to support discussion of potential refinements, additions, amalgamation, or refuting of theories from the realist review. Tables were created to show existing CMOs, alongside notes, reflections, and emerging data insights with colour coding of the data source. Through an iterative process which included multiple rounds of discussion, the final refined CMOs were numerically labelled under each programme area, with quotes and data extracts to illustrate how theoretical thinking developed.

### 2.6 Rigour Related to Data Collection and Analysis

The development and refinement of focus group materials and topic guides was supported by an expert steering group including parents of children with SSD, SLTs and a clinical psychologist. A pilot focus group was held with five SLTs, and minor modifications were made. Reflective logs were kept when conducting, transcribing, and analysing focus groups to support reflexivity.

During analysis, two focus group transcripts (28.6%) were read independently in full by JT and EP to support validation. They highlighted and added comments to transcripts and created a reflective log of how data related to existing CMOs or added new insight. Comments and reflections were discussed with JT and EP, corroborating consensus on the thinking emerging from the data. Regular research team meetings helped validate new CMOs. A summary of key findings was shared with 20% of participants for member checking. Clear documentation of the data collection and analysis process was maintained to ensure transparency. Extracts and quotes demonstrate how data informed CMO refinement.

## 3 Results

### 3.1 Main Findings

Realist logic has been used to elicit insight into how an intensive, digital parent‐implemented intervention works for children with SSD, and which contexts impact outcomes. The final output in realist methodology is the refinement of exploratory theoretical thinking to help understand what works, for whom, why, how, and in which situations (Jagosh et al. 2015). This theoretical thinking is depicted as a set of refined CMOs. CMOs developed through an earlier realist review (Leafe et al. 2025) have been further refined in this evaluation through insight gathered from key stakeholders. CMOs are informed by primary data and retroductive theorising, which involves moving between deductive and inductive reasoning and author insight to generate causal explanations (see Table 1 for key terms used in realist methodology) (Jagosh et al. 2015; Greenhalgh et al. 2017a).

In this realist evaluation, the journey many parent/carers take when participating in digital parent‐implemented interventions emerged as a key line of thinking, including (1) readiness to engage, (2) implementation of the intervention, and (3) sustaining momentum. The CMOs linked to the three aspects of the parents’/carers’ journey are summarised in the results below, with choice extracts of data and models to illustrate theoretical thinking. The full set of CMOs that support the results and the theoretical thinking outlined below can be accessed via Ulster University's Research Portal at DOI (Leafe et al. 2025), which includes further quotes for each CMO^2^. The reader will be signposted to the CMOs throughout the results for further evidence of the development of theoretical thinking.

**FIGURE 1:**
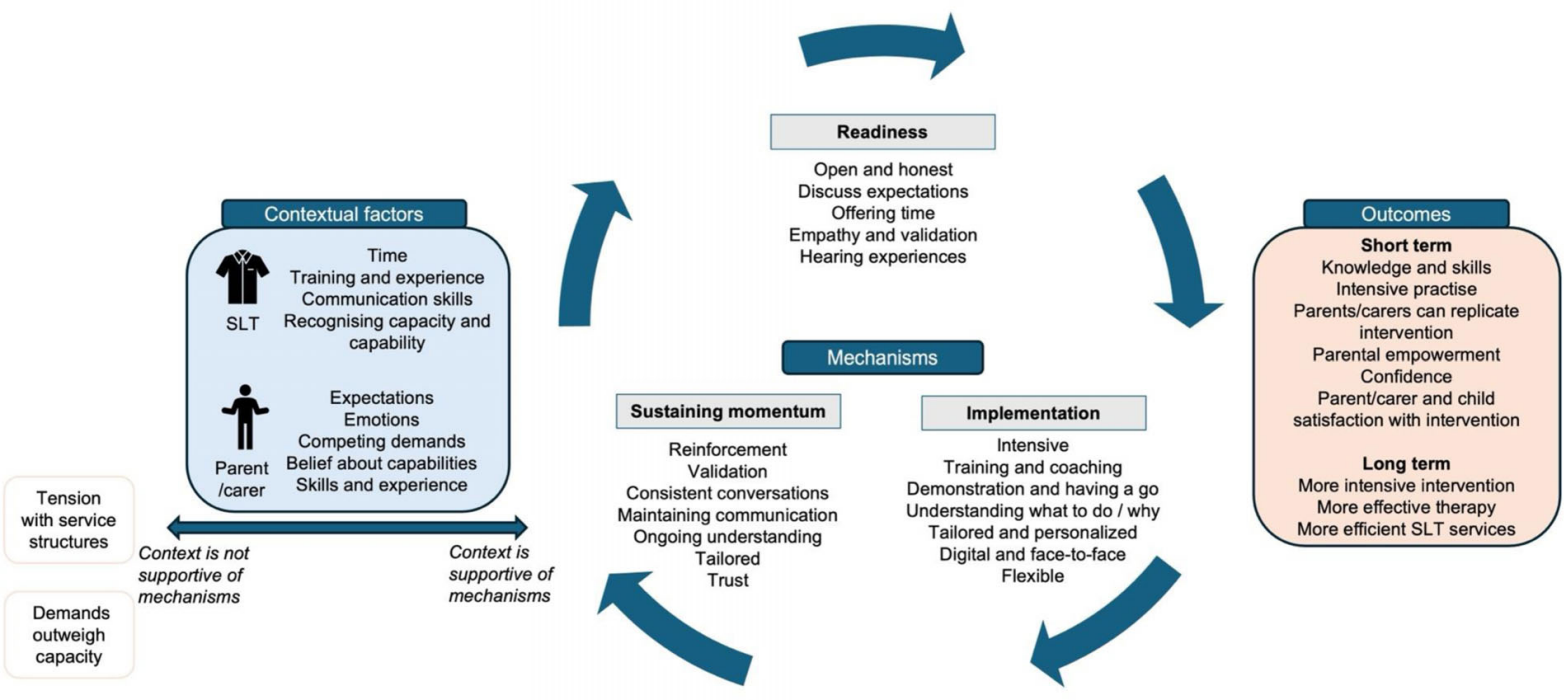
Final explanatory model showing the how contexts interact with mechanisms to produce outcomes in digital, intensive, parent‐implemented interventions for their child with SSD. SSD, speech sound disorder.

Existing theory has been applied across the journey stages to add depth to thinking about how it works and why. Theories around self‐efficacy, empowerment, adult‐learning, and parenting styles were relevant to parents/carers in relation to readiness, implementation, and momentum to engage. For SLTs, the theory of teaching styles was relevant across each aspect of the journey. These middle‐range theories are linked to findings in the discussion. The final programme theories have been illustrated in Figure 1, showing contextual factors impacting the SLT and parent/carer, the mechanisms involved across the journey, and potential outcomes of the intervention.

#### 3.1.1 Readiness: What Is Needed for Parents/Carers to Engage?

Parents/carers described the need to feel motivated, willing, and confident in their ability to engage in intensive digital parent‐implemented intervention. SLTs have a role to play in building a trusting, therapeutic relationship with parents/carers, to empower them and create foundations for successful teamwork and partnership from the outset. The communication and interaction skills used by SLTs, often implicit, can impact on parent/carers investment in the process, referred to by stakeholders as ‘buy‐in’ (P2, SLT; P9, parent/carer; P17, SLT; P20, SLT). Children with SSD will likely require sustained, intensive intervention, supported through direct SLT appointments and at home by parents/carers. Therefore, buy‐in and readiness to engage are imperative. The related CMOs that have informed the following areas are noted throughout.

##### 3.1.1.1 Open and Honest Conversations

Stakeholders described how dedicating time to an open and honest conversation at the start of the journey helps create shared understanding and build a sense of partnership (CMO 2.5). Discussing the potential journey and intervention roles helps set expectations and accountability for all involved, and creates a platform for parent/carers to ask questions—a helpful starting place for open, honest communication. In these conversations, parents/carers are empowered when their importance and expertise are emphasised.

‘I guess just to know your place in it all, you know, to know that you are a link in the chain.’ (P27, parent/carer)

Allocating a whole session to this conversation could create more sustained motivation, investment, and engagement in parent‐implemented intervention.

##### 3.1.1.2 Empathy and Validation

Parents/carers can experience difficult emotions when their child needs support for SSD, including worry, frustration, overwhelm, or vulnerability to criticism. Their level of concern, view towards professionals, or past experiences of services impact their mindset at the start of their journey. This worldview affects how a parent/carer may initiate, accept, or respond to support or the suggestion of parent‐implemented intervention (linked to CMOs 2.1 and 2.5 in the full set of CMOs, pages 3 and 6).

‘I think sometimes parents think speech therapy is like going to physio or going to the dentist where they take their child and you fix them and they just have to be the one that brings them and takes them home again, and they don't actually realise… they have a part to play in speech therapy.’ (P23, SLT)

**FIGURE 2:**
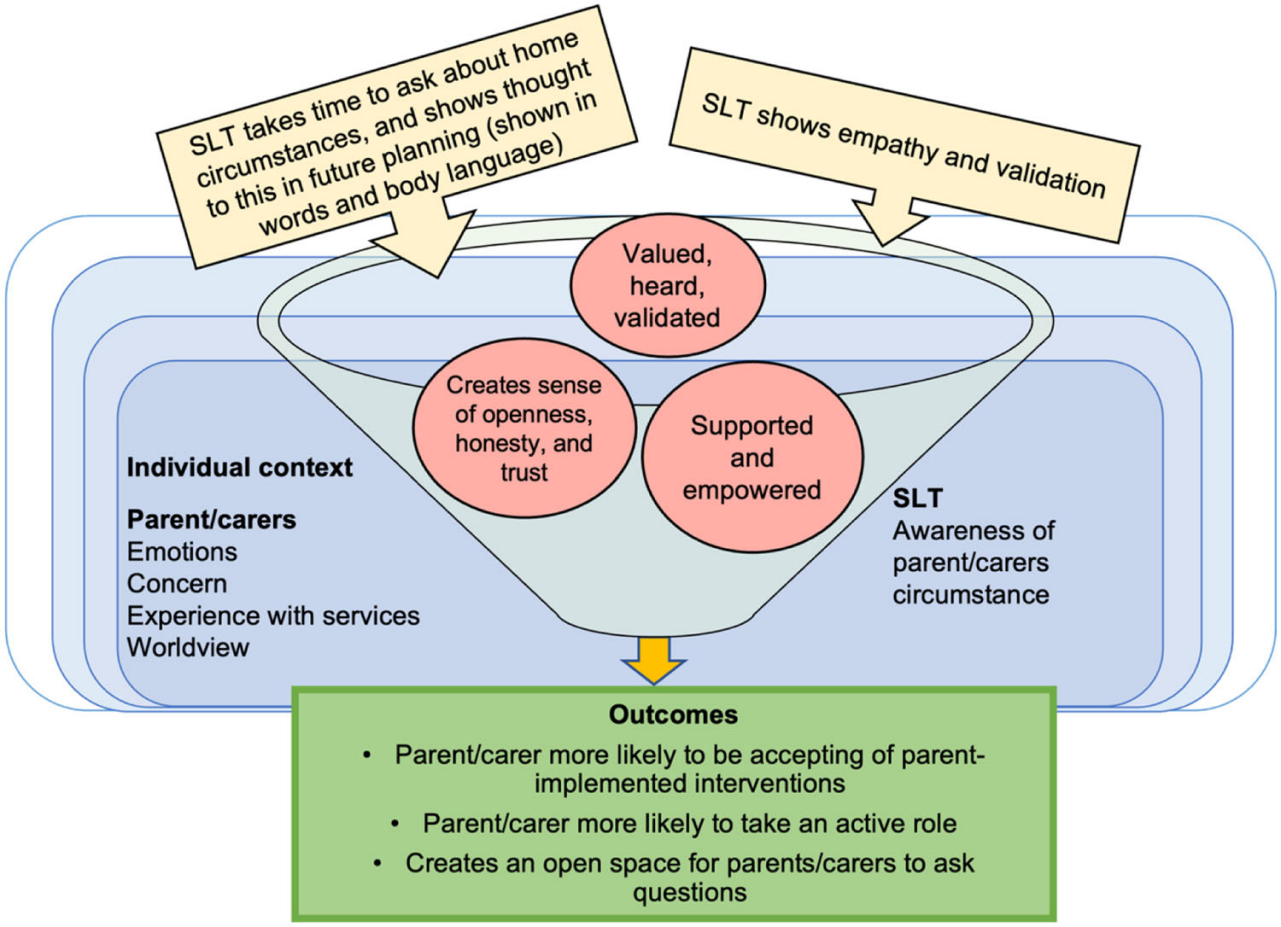
CMO 2.1: Providing opportunities to be open and honest leads to a trusting relationship between SLT and parent/carer (pink circles show parent/carer responses). SLT, speech and language therapist.

SLTs drew on the importance of being aware of the parent/carer's perspective and past experiences and recognising their own position as a professional. Approaching parents/carers with empathy and understanding helps parents/carers feel validated, valued, and heard (CMO 2.1, shown in Figure 2).

‘Sometimes we're the only people that they can confide in and and tell things so it's being very aware of that… we are the people for the parent… we feel like we have to be there for them.’ (P15, SLT)

SLTs demonstrating they have time to listen to parents/carers and recognising their needs, for example through body language, may help them feel supported and empowered to adopt a more active role (CMO 2.1).

‘…non‐verbal cues like looking at the clock… you think ‘okay well you know we just have to, we'll just go and and let her do her job’… I guess things things like that really come into play here um within the overall experience.’ (P22, parent/carer)

##### 3.1.1.3 Service Contexts

**FIGURE 3:**
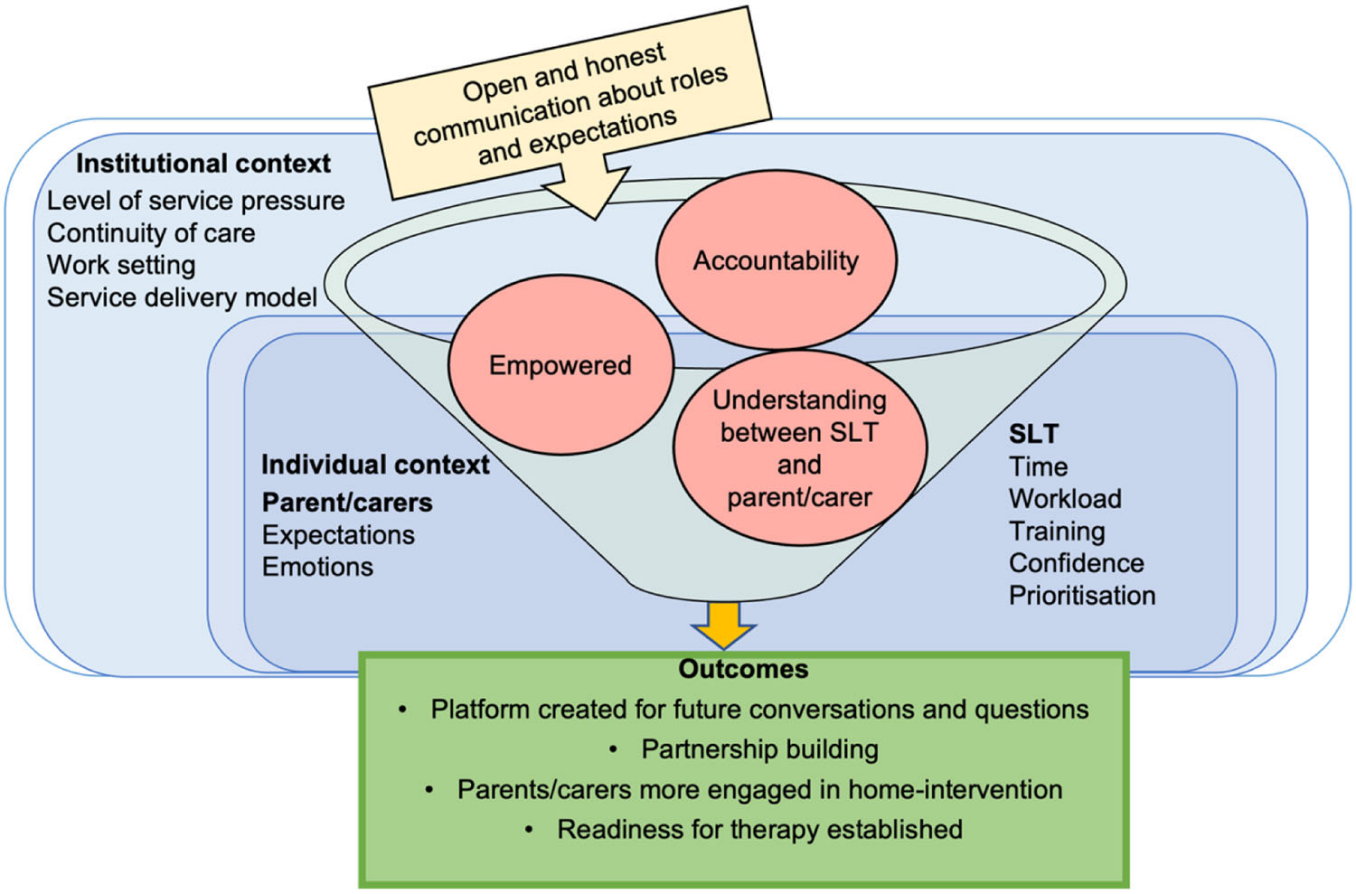
CMO 2.3: Individual and service level contexts impact on SLT confidence and views towards parental involvement and 2.5 A conversation about roles at the start of therapy helps develop shared expectations and role accountability. SLT, speech and language therapist.

There is tension between SLTs wanting to spend time building relationships but feeling constrained by service pressures and continuity of care (see CMO 2.3, page 5 in the full set of CMOs). Service structures or settings can enhance communication with parents/carers (e.g., specialists who can offer more intensive therapy), or make it more challenging (e.g., schools with limited parent/carer contact, or reduced staffing and high clinical demands). The level of SLT training, confidence, and experience working with parents/carers impacts how much they involve them in intervention (as seen in Figure 3). In pressured circumstances, it can be difficult for SLTs to prioritise relationship building and parent‐implemented interventions over other demands on time, leading to reduced opportunity for parents/carers to be supported to implement home‐intervention.

‘I'm more defensive now, I'm having to explain [to them] why you're not getting therapy… now I feel like I'm rushing through things and that that social connectivity is really important I think… with service pressures at the minute… I don't have time to build that same relationship.’ (P15, SLT)

‘It comes down to time in the sense that if you haven't had time to build that relationship, you are seen as this person that just sort of flies in, does the therapy, flies out again.’ (P14, SLT)

##### 3.1.1.4 Hearing From Other Parents/Carers

Both stakeholder groups reflected on the support parents/carers could feel from hearing from those who have experienced a similar journey (CMO 3.5). It could help them understand the potential road ahead and reduce isolation. Hearing from someone with first‐hand experience about how home‐practice could help would be motivating.

‘What I would love to see is maybe another parent speaking and saying “here's a success story, you know we're at the end of our journey…” you know just to give you hope, and, to understand that if you do put this work in, I know obviously all the outcomes are different, but you know as a parent it's it's frightening… if you could see somebody that's come out the other end… [that] definitely [would have] motivated to me to keep going.’ (P26, parent/carer)

##### 3.1.1.5 Readiness Identification

SLTs and parents/carers agreed that an intensive, digital, parent‐implemented intervention may not be appropriate for everyone. Part of the SLT's role is understanding parent/carer circumstances and competing demands and how these impact capacity to take part. If being asked to engage outweighs parents/carers actual or perceived capacity and capability, they may feel burdened. Through open conversations, SLTs can ascertain if the family are at a time and place to engage in digital, intensive, parent‐implemented intervention (linked to CMO 2.1).

‘Sometimes I will have a conversation with a parent you know if it's very obvious that there's maybe an awful lot going on and just ask them you know “is this a good time for therapy, do you feel you you could actually take this approach on board or would you prefer to just you know set aside from [therapy] and we'll come back to this?” because if it's not their priority you know it's it's not a waste but you know it's not the best use of their time…’ (P23, SLT)

#### 3.1.2 Implementation

Once established if digital, intensive, parent‐implemented intervention is appropriate, implementation needs to be tailored and flexible to individual contexts to increase empowerment and success. In CMO development, it was clear that the digital element of the intervention provides a platform for the implementation of intensive, parent‐implemented intervention. While it offers some unique resources, many of the unobservable, underlying intervention mechanisms of the intervention (as outlined below) exist irrespective of the delivery mode.

##### 3.1.2.1 Training and Coaching

Parents/carers need appropriate, personalised, timely coaching and training from the outset of the intervention and throughout (related to programme area 3 in the full set of CMOs). All parents/carers start with different experiences, knowledge, and skills. Training needs to be tailored to build on these skills, so parents/carers feel confident and capable to deliver intervention at home. Parents/carers need information on what to do, opportunities to observe demonstrations of activities and techniques, and encouragement to have a go to help them visualise, learn, retain, and replicate potentially abstract concepts (linked to CMO 3.2, page 8 of the full set of CMOs).

Different modes of information and communication help embed adult learning, such as written information, pictures, drawings, demonstrations, practical resources, videos, or recordings. Access to relevant information at suitable times, such as messages reinforced through a digital platform, helps parents/carers remember what to do in the context of busy lives.

‘I've had parents saying, “could I record you saying that part?”… because they say ‘I think I know what I'm doing until I go home and then I forget the way you did it.’ (P2, SLT)

**FIGURE 4:**
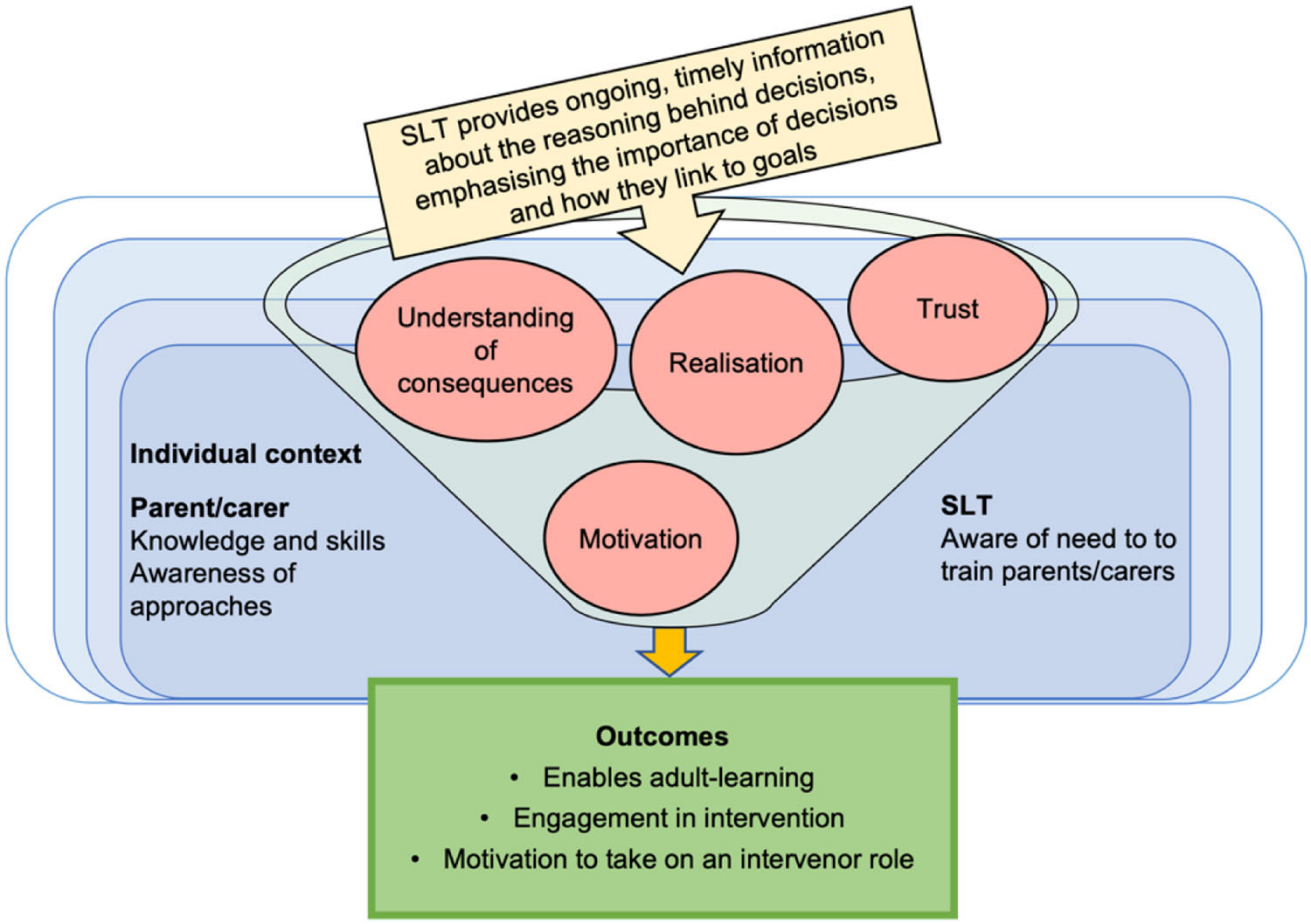
CMO 3.1: Providing ongoing information about the reasoning behind decisions increases parent/carer motivation to engage.

Parents/carers need to hear why the intervention is important, including the reasoning behind decisions (for example, targets, activities, dosage, parent/carer involvement) with information about how it will help their child progress towards their goal (e.g., more repetitions may mean faster progress). Understanding the importance and consequence of decisions builds trust in the process and creates internal motivation to engage (CMO 3.1, as illustrated in Figure 4).

‘I think it helps get a little bit more buy‐in from the parents because they're seeing “okay there is a‐ an outcome from this, that there is a reason why we're investing time into this and there's a reason why it's not just happening in the therapy sessions.”’ (P17, SLT)

‘…you want the best for your child so of course you want to do everything for them, but if they understand the impact that it's going to have … and the reason for you know doing it… one hour a week isn't going to be enough, so you know we must do this every week and you will see great you know results.’ (P9, parent/carer)

##### 3.1.2.2 Balancing the Role of Parenting and Delivering Intervention

Parents/carers reflected that their relationship with their child has more emotional weight than between their child and their SLT, teachers, or other family members who might be more detached. Children may perceive parent/carers as less authoritative, and may be more inclined to want to please those with less attachment, or less likely to say no.

‘… maybe toddlers can smell fear [from the parent] I don't know what it‐ what it is, but you know, I think they just need to have that very you know, when you're in that session they need to have that very authoritative, you know lead on it.’ (P22, parent/carer)

Parents/carers reported finding it helpful to replicate the SLT's techniques and behaviours in activities at home, such as mimicking their confidence. Some parents/carers felt their child may be more willing to engage if they perceive as more like their SLT (CMO 4.2).

‘It's a bit like fake it till you make it and you're trying to remember, you know the tone that the SLT has used and the tactics that they've used and and um doing your best to mimic those … if you let that slip and that kind of authoritativeness slip, you know that's their focus gone sometimes … I think the confidence and showing them that, you know you're you're leading… like the SLT, it is needed to keep them, to keep their focus.’ (P22, parent/carer)

This may be dependent on their rapport with their SLT, their parenting style, or how comfortable children feel with their parent/carer changing role.

##### 3.1.2.3 Tailoring Intensive Intervention

Intervention intensity needs to be optimal for the child. Depending on the severity of their SSD, therapy practice should be at least two to four times a week, with over 70 repetitions of targets per session. Therapy should continue for as long as it is still beneficial. Targets and intervention approaches need to be evidence‐based and appropriate for the nature of SSD (CMO 1.4). These factors facilitate deep, enhanced learning to support children's progress (linked to CMOs 1.1, 1.3, and 1.4, pages 1–3 in the full set of CMOs).

To support more intensive intervention at home, SLT flexibility and problem solving with parents/carers helps integrate practice into family routines. It helps the parents/carers feel involved and heard, gives them a sense of choice, and can make home‐practice more achievable and generalisable to improve outcomes. Tailored activities feel achievable, which builds parents/carers optimism about their abilities to practise, increasing confidence and potentially reducing overwhelm and burden. This links back to open communication developed at the start of the journey (see CMO 2.1 and 2.2).

‘…if they were saying like it's just very rushed with three or four children at home and then I started to problem‐solve things like “well what about this, could you try this instead? What about this be a good time for you?” and then they can come up with their own ideas, and then I just think it motivates them a lot more to think ‘well maybe this will work if we try this or if we try this instead or set aside this time’ and then it sort of empowers them to think ‘well then we could do this.’ (P26, SLT)

**FIGURE 5:**
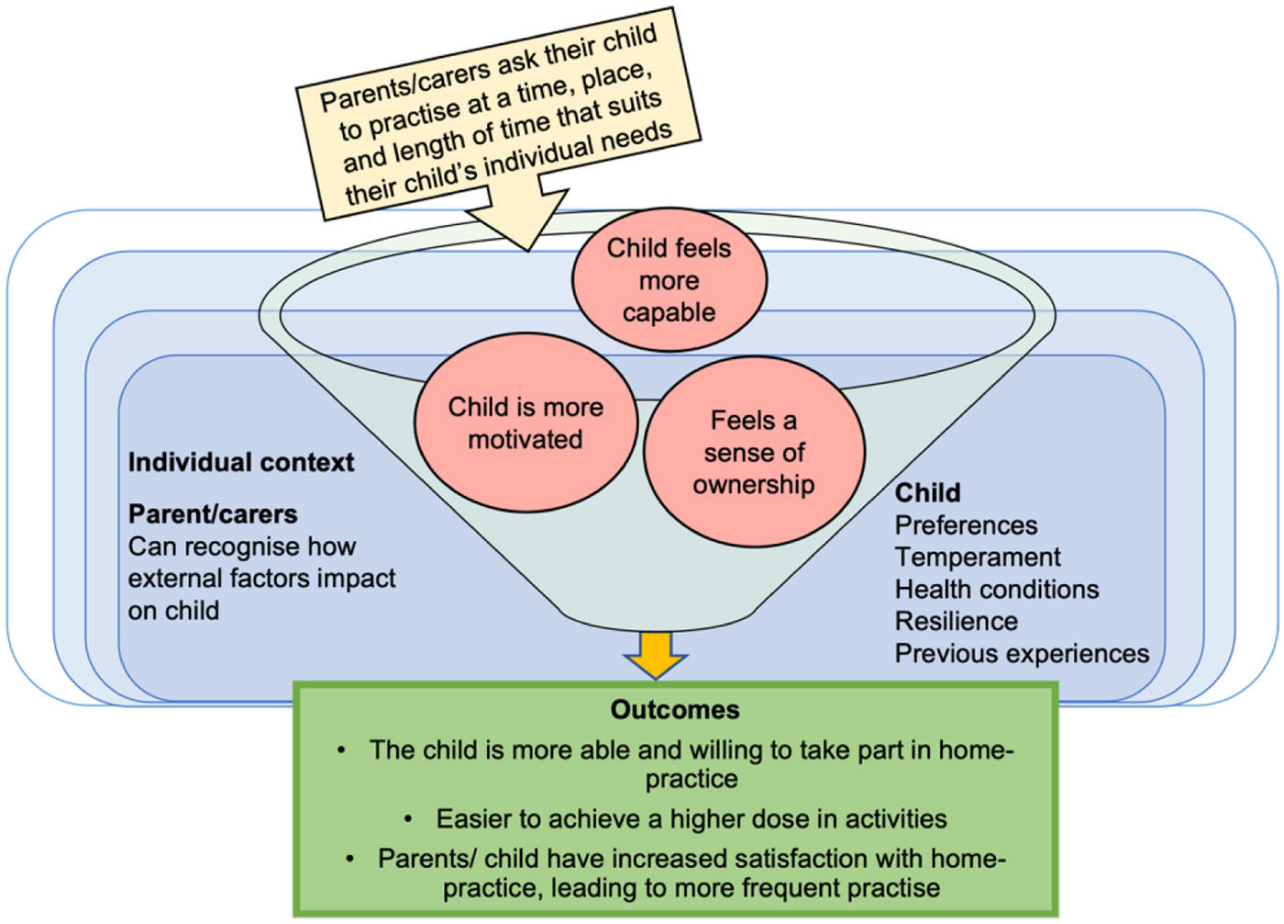
CMO 5.1: Personalising home‐practice to suit the child's individual context impacts child participation.

Parent‐implemented intervention provides a unique opportunity for children to receive therapy when it suits them, which is less likely with SLT clinic appointments. Practising at a time, place, and duration to suit their child's temperament, energy, and interests will increase their capability and willingness to engage and focus, as shown in Figure 5 (CMO 5.1). Some parents/carers may need support to recognise this.

‘… when they're tired it requires a lot more effort and it's harder to concentrate it's harder to do and just less motivated generally like all of us.’ (P8, SLT)

##### 3.1.2.4 Personalisation

Customisation of therapy activities, such as games or characters, helps motivate children to engage in digital, parent‐implemented interventions (CMO 5.4). Customised activities are more likely to meet their interests, be meaningful and relatable, and feel fun because of associations with games they enjoy. This helps children engage in more intensive practice (CMO 5.4) (see full set of CMOs, page 14 for further detail and supporting quotes). Digital games may also be positively associated with enjoyable activities, and the mobility of phone‐ or tablet‐based devices allows practice in different locations, creating more practice opportunities and facilities to adapt to children's preferences (see CMO 5.6 in full set of CMOs, page 15).

Task difficulty is noted to impact child‐engagement and learning, and therefore needs to be personalised. Targets and games need to be a combination of achievable and challenging, so they feel success and build on existing knowledge and skills, known as challenge point (Guadagnoli and Lee 2004). Balancing these factors increases satisfaction in home‐practice and optimises learning but depends on the level of the child's awareness and resilience (CMO 5.2).

‘… if it's either too difficult or too easy it becomes demotivating… they need it to be at just the right sort of level of challenge to keep it‐ to keep it interesting… if it's too easy um that there isn't that same sense of pride when they get it and if it's too difficult or too hard they get demotivated very quickly… they don't they don't want that negative feeling again.’ (P8, SLT)

#### 3.1.3 Sustaining Momentum

The digital tool and SLT both play a role in supporting parents/carers and children to sustain their momentum with implementing intensive parent‐implemented intervention.

##### 3.1.3.1 Reward

Stakeholders described different reward functions in sustaining children's motivation to practise at home (see CMO 5.5 in the full set of CMOs for further detail and quotes). Clear and visible reward increments are incentivising, for example, building up small tokens for a bigger reward. Having a choice of rewards increases motivation.

‘If they get it all right you know they get another stamper on their chart and then when they get this chart finished, they have a prize … that idea that they're working towards something‐ something big that‐ that's gonna work.’ (P6, SLT).

**FIGURE 6:**
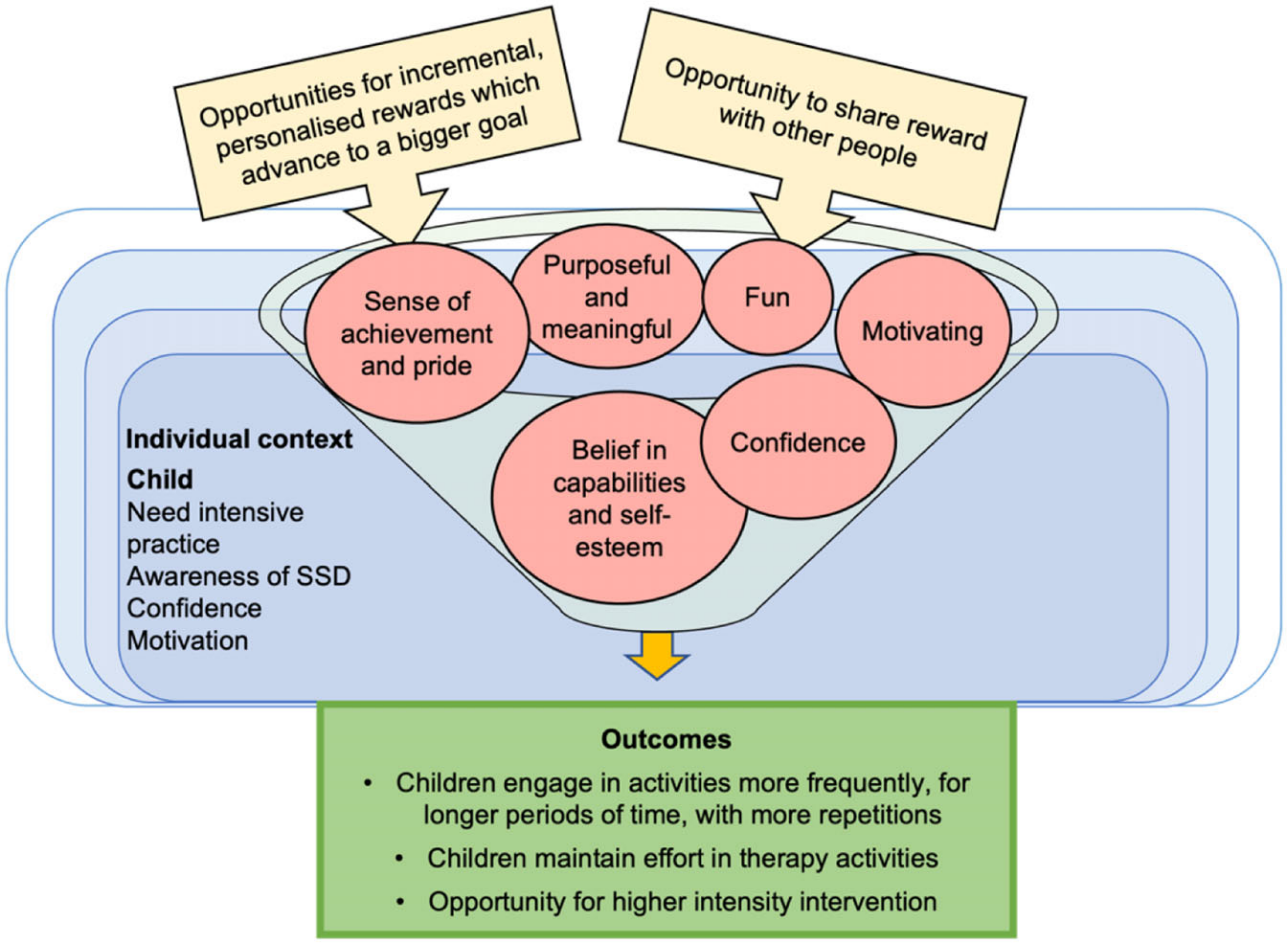
CMO 5.5: Personalised reward system towards an end goal increases child engagement.

Some children will feel pride and success from sharing these achievements with other people, which increases confidence and self‐esteem and can provide rewarding social connection and validation. Children will be motivated to maintain feelings of success, increasing momentum to engage in speech activities more frequently, for longer periods of time, with more repetitions, leading to higher intensity of intervention (linked to CMO 5.3; CMO 5.5, illustrated in Figure 6).

‘It's very reinforcing because they get um that double impact of that good feeling, so they're getting that good feeling when they achieve and then when other people acknowledge their achievement they get it again and it just makes them more likely want to get that feeling again.’ (P8, SLT)

##### 3.1.3.2 Maintaining Communication

Both stakeholder groups reported that gaps between SLT contact reduce momentum (CMO 1.1). Some SLT services follow a model of offering a 6‐week block of sessions followed by a break. Parents/carers and SLTs described how maintaining communication, monitoring, and training during break periods through a digital tool would build confidence and motivation.

‘… I had forgotten all of the skills I had I had built up as well … you do lose all momentum on it both as a‐ my child and myself… because of the I'd say in the time delays.’ (P22, parent/carer)

‘…perhaps you might get a parent potentially… [who] is just is starting to become confident in doing a particular um you know doing a particular approach, in working on a particular sound or cluster or whatever, but then the break happens and it's kind of back to square one um so I suppose that I I feel that that potentially um could have had an impact on confidence.’ (P5, SLT)

##### 3.1.3.3 Timely Reinforcement

All participants reported that timely feedback and positive reinforcement for parents/carers, when they are open to this, helps them reflect on and either repeat or change their actions. This maintains motivation and reduces anxiety because parents/carers feel confident in what they are doing, increasing the success and effectiveness of home‐practice (CMO 3.2).

‘… if you're not sure on how effectively you're doing it, it really zaps your motivation for for keeping going, so I think that's maybe something to think about too when it comes to that relationship with the SLT.’ (P22, parent/carer)

‘You need to bring the parent through as well as the child… even saying you know like my one said, say “Mum you have done a lot of work this week, Mum, that's great” … sometimes it's just getting that … “oh you're doing well too” it's like ‘oh right ok, let's keep going.’ (P9, parent/carer)

##### 3.1.3.4 Consistent Communication About Home‐Practice

**FIGURE 7:**
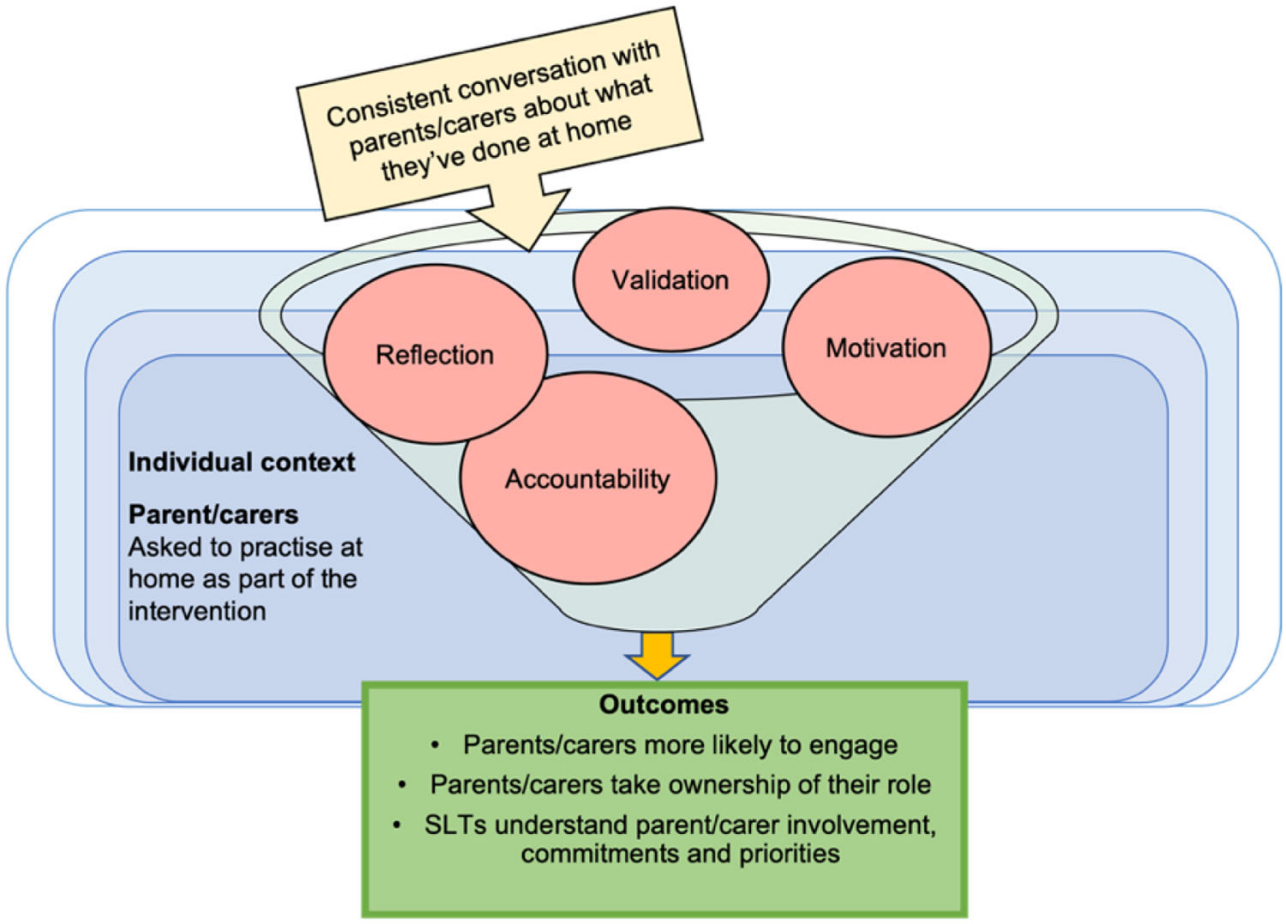
CMO 3.4: A consistent conversation about home‐practice creates parent/carer accountability and ownership over roles.

Consistent communication between the SLT and parent/carer about home‐practice reinforces accountability, ownership, and partnership working (see CMO 3.4). If parents/carers know they will be asked about home‐practice, they feel responsible to practice. Asking parents/carers also supports future tailoring of activities or reassessment of readiness. This is shown in Figure 7.

‘If they knew that like a standing you know agenda point if you like of the‐the session was that the SLT says “how did your home practice go?” and you have a ring‐fenced five‐minute discussion on that, you would do your homework, parents to at least give it a go because… they're accountable.’ (P22, parent/carer)

##### 3.1.3.5 Ongoing Understanding

Honest and open communication continues to act as a mechanism throughout intervention. SLTs need to create a safe space for parents/carers to say if home‐practice has gone well or not, to increase alliance, partnership, and maintain engagement (linked to CMOs 2.1 and 3.4).

‘I think in an ideal world you get to the point where the parent is comfortable enough with you to say “we had a nightmare of a week we didn't get near it’ and you're gonna go ‘I understand… that's okay you know we've all had them”.’ (P18, SLT)

Some SLTs stated it is important to show their own vulnerabilities and be open about themselves to build trust and help parents/carers feel comfortable to ask questions and share concerns. This communication continues to support activity tailoring, making parent‐implemented intervention more successful (CMO 2.1; 2.2)

## 4 Discussion

This realist evaluation has uncovered key mechanisms, contexts, and outcomes of a potential novel intensive, digital, parent‐implemented intervention for children with SSD. It considers perspectives of parents/carers and SLTs, building on findings from the earlier initial realist review (Leafe et al. 2025). Digital, parent‐implemented interventions are most likely to be successful when there are three core components: readiness to engage, supported implementation, and facilitation of sustained engagement.

Readiness depends on balancing capacity and demand. SLTs investing time to develop trusting, therapeutic relationships with parents/carers creates the foundations for future successful outcomes. However, competing demands within SLT service structures often reduce the capacity to invest time in building strong therapeutic relationships with parents/carers. Successful implementation also relies on flexibility and tailoring to individual contexts, with parents/carers feeling empowered through personalised coaching, training, and timely support. Opportunities to observe, practise, and replicate behaviours and techniques are key for some parents/carers. Children with SSD need to receive optimal, tailored intervention intensity, with targets at the right challenge point for their skills. Sustained implementation requires reinforcement and communication, with rewards, digital games, and customisation helping maintain home‐practice. The findings from this evaluation show that digital tools may facilitate behaviour change mechanisms but must be used alongside an SLT to promote intervention mechanisms.

Our findings can be positioned within a wider understanding of complex health interventions. Pawson (2013) notes that one aspect of intervention complexity is that individuals respond differently based on their worldviews. He describes this in terms of the volitions of those receiving the intervention, that is, the cognitive process a person goes through to make choices about their actions. Our realist evaluation highlights the need for continuous support for parents/carers and children throughout the SLT intervention journey to help parents/carers and children from different starting points, addressing individual contexts and mechanisms at each point, such as skills, tools, or resources.

Parental empowerment, confidence and self‐efficacy are crucial at each stage. In self‐efficacy theory (Bandura 1997), empowerment relates to a person's self‐perception of their competence and goal attainment. Empowerment in healthcare requires a participatory way of thinking, where patient‐centeredness, personalisation, customisation, trust, and open communication are adopted (Halvorsen et al. 2020). Empowerment involves the development of motivation, skills, and knowledge to participate in making choices (Fumagalli et al. 2015). In digital, parent‐implemented interventions, empowerment can be supported through tailoring intervention to the individual (including training and coaching), building trusting, therapeutic relationships, and creating space for open and honest communication. This study provides deep insights into mechanisms that foster empowerment and self‐efficacy for successful engagement in the intervention.

Our findings support the importance of the therapeutic relationship between SLTs and parents/carers, as reported in other studies (Davies et al. 2017, 2019; Watts Pappas et al. 2016; Sugden et al. 2019; Klatte et al. 2020). As in Klatte et al. (2020), communication, trust and contextual factors such as parental expectations, capacity, and capabilities impact collaborative practice. However, our evaluation also explored aspects involved in digital, parent‐implemented interventions and how they develop and facilitate collaboration. We offer new insights into interventions for children with SSD, specifically around the journey a parent/carer may take, and the specific mechanisms required for this group, including coaching on techniques, and optimal intensity to support effective outcomes. This is an area that has not been previously explored using realist methodology.

This study shows that developing a trusting, supportive relationship is essential for later outcomes. Whilst SLTs recognise the importance of building these relationships, existing NHS service structures often hinder realisation in clinical practice. Our findings show that investing time in this can impact the experiences of all involved and the future effectiveness of therapy. In a context where NHS services are stretched and traditional models of service delivery offer blocks of therapy with gaps between blocks for children with SSD, providing the recommended intensity of intervention can be challenging for SLTs. It was recognised that digital parent‐implemented interventions may help increase practice intensity when specific mechanisms, including those associated with coaching, are implemented.

The findings have illuminated thinking around the implicit skills that SLTs use in coaching parents/carers. Teaching styles and adult‐learning styles are closely related. Like the reflections of participants in this study, learning is most effective when teaching complements a person's preferred way of learning. Deeper into adult‐learning theory (Knowles et al. 2015), this study shows how training and coaching from an SLT, supported through a digital tool, needs to explain why decisions have been made and why the learning and involvement are important. As in adult‐learning theory, parents/carers reported that hearing from other experienced parents/carers about how intensive practice leads to end goals and success would be motivating. They are more likely to engage in learning when coaching and training is tailored to their existing knowledge and skills, with information relevant to them. Adults need to be ready to learn (Knowles et al. 2015), which links to the first stage of the journey identified in this study.

New insights were gained regarding parent/carers feeling that they need to mimic SLT behaviours for successful home‐practice. Parents/carers spoke about needing to be authoritative to help their child take part. Baumrind (1971, 1991) and Maccoby and Martin (1983) studied parenting styles, identifying styles with different levels of demand or responsiveness to their child (authoritative, authoritarian, indulgent, and neglectful or uninvolved). Kuppens and Ceulemans (2019) built on this work and established four parenting styles in their work, labelled as authoritative, positive authoritative, authoritarian, and uninvolved. A positive authoritative parenting style shows involvement, reinforcement, sets rules, and uses behaviours to encourage independence and promote wanted behaviours. Authoritative parenting adopts the same positive approaches but also uses actions to discourage unwanted behaviour (e.g., discipline, punishment, or ignoring). Authoritarian styles show more punishment and discipline and less positive parenting than average, while uninvolved parenting styles show below‐average levels of warmth, involvement, discipline and punishment.

Parents/carers reported needing to adopt the therapist's approach when delivering home‐intervention, which some described as more authoritative. SLT participants described certain positive authoritative behaviours when talking about their therapy interactions, for example, warmth and responsiveness, but also demonstrating high support, reinforcement, and clear expectations (Kuppens and Ceulemans 2019). If a parent/carer is typically not positive authoritative, mimicking the SLTs approach may require a shift in parenting style. At different points in the journey of intervention, children may require different styles according to their needs. For example, depending on personality or awareness of their SSD, they may require lower demands, and higher responsiveness to build their confidence and motivation to take part. Too big a shift between their typical parenting style and the style parents/carers adopt when implementing intervention may be challenging for children and parents/carers. This would benefit from further exploration in future empirical studies.

### 4.1 Strengths, Limitations and Future Directions

This study provides new insights by capturing the views of parents/carers and SLTs using a realist methodology approach. We gathered perspectives from a range of SLTs with varied experiences from NI and across the United Kingdom. Realist methods do not aim to be generalisable or replicable; however, careful planning of data collection and analysis has supported the rigour, trustworthiness and credibility of the findings. While the number of parents/carers involved was smaller than anticipated, the captured data was rich and insightful. Although programme theories have been developed about the child, the child's voice was only able to be indirectly captured through the SLTs and their parents/carers. Future research should include the child's perspective, as advocated by McCormack et al. (2022).

### 4.2 Recommendations for Clinical Practice

The key clinical recommendations emerging from findings have been summarised in Table 4.

**TABLE 4:** Table to show the key clinical recommendations for implementing in practice.

Key recommendations for SLTs in clinical practice	Stage of journey		
	Readiness	Implementation	Sustaining momentum
1. Dedicate time (preferably a whole session at the start of intervention) to develop open and honest communication with parents/carers. Discuss expectations and plan your roles.	X		
2. Establish the readiness of parents/carers to take part through talking about home‐life (e.g., time, commitments, skills, access to digital device, demands).	X		
3. Tailor home‐practice to meet the needs of the child and family. Problem‐solve with parents/carers about how activities can be incorporated into existing routines.	X	X	
4. Emphasise the expertise of the parent/carer in your conversations and feedback.	X	X	X
5. Provide coaching and training for parents/carers about what to practise at home and how to do it, including demonstrating, encouraging them to have a go, and offering feedback to help them reflect on their implementation of activities.		X	X
6. Explain why decisions have been made and how activities, learning, and targets link to their goals.		X	X
7. Set specific times to ask parents/carers about their home‐practice between sessions.		X	X
8. Support parents/carers to identify times that their child is most likely to be able to engage, for example, time, place, and length of time that suits their temperament, energy, and interests.		X	X
9. Create opportunities for personalised, incremental rewards, including social rewards and reinforcement.		X	X
10. Incorporate digital tools to incentivise children, monitor home‐practice, and train parents/carers alongside face‐to‐face support. Different modes of communication, for example, videos, written information, sound clips, pictures, and infographics, help accessibility to learning.		X	X

## 5 Conclusion

Digital, parent‐implemented interventions for children with SSD can support the delivery of intensive, evidence‐based intervention. This intervention will be more suitable for some parents/carers and families than others, a factor which needs to be identified and recognised by SLTs. Successful delivery of digital parent‐implemented interventions requires different mechanisms at different time points, suited to the individual's contexts, to help support readiness, implementation, and sustained engagement. In NHS services where demands on clinicians are high, SLTs need to be supported to invest and dedicate time to developing relationships with parents/carers. An appropriate digital tool has the potential to provide a platform for delivering customised, tailored, motivating activities for home‐practice. However, underlying intervention mechanisms identified in this evaluation are needed for the digital tool to be effective. Future work with parents/carers, SLTs, and children could help co‐produce the key requirements of a digital tool based on the underpinning theoretical thinking developed in this evaluation.

## Ethics Statement

Ethical approval for the study was obtained from NHS Research Ethics Committee (Reference: 22/PR/0651; IRAS Project ID: 314024).

## Conflicts of Interest

The authors declare no conflicts of interest.

## Data Availability

The data supporting the findings reported in this paper are openly available from Ulster University's Research Portal at DOI (Leafe et al. 2025)

## Endnotes

1 The TDF (Atkins et al. 2017) is an overarching framework that amalgamates behaviour change theories, integrating key constructs, grouped into 14 domains to inform evaluation and development of interventions. For example, the *belief about capabilities* domain links to constructs such as self‐efficacy, and empowerment. These constructs were used to expand and interpret data from participants.

2 The CMOs are organised into the same five overarching programme areas as the realist review (Leafe et al. 2025), but readers of the realist review may note that they are in a different order in this publication.
